# Biological Contamination Prevention for Outer Solar System Moons of Astrobiological Interest: What Do We Need to Know?

**DOI:** 10.1089/ast.2018.1996

**Published:** 2019-07-29

**Authors:** Petra Rettberg, André Antunes, John Brucato, Patricia Cabezas, Geoffrey Collins, Alissa Haddaji, Gerhard Kminek, Stefan Leuko, Susan McKenna-Lawlor, Christine Moissl-Eichinger, Jean-Louis Fellous, Karen Olsson-Francis, David Pearce, Elke Rabbow, Samuel Royle, Mark Saunders, Mark Sephton, Andy Spry, Nicolas Walter, Robert Wimmer Schweingruber, Jean-Charles Treuet

**Affiliations:** ^1^ Research Group Astrobiology, Radiation Biology Department, German Aerospace Center (DLR), Institute of Aerospace Medicine, Köln, Germany.; ^2^ GEMM—Group for Extreme and Marine Microbiology, Department of Biology, Edge Hill University, Ormskirk, United Kingdom.; ^3^ Department of Physics and Astronomy, Astrophysical Observatory of Arcetri, National Institute for Astrophysics (INAF), Florence, Italy.; ^4^ Science Connect–European Science Foundation (ESF), Strasbourg, France.; ^5^ Department of Physics and Astronomy, Wheaton College, Massachusetts, Norton, Massachusetts.; ^6^ Committee on Space Research (COSPAR), Montpellier, France.; ^7^ Space Technology Ireland Ltd., Maynooth, Ireleand.; ^8^ Department of Internal Medicine, Medical University of Graz, Graz, Austria.; ^9^ Faculty of Science, Technology, Engineering & Mathematics, School of Environment, Earth & Ecosystem Sciences, The Open University, Milton Keynes, United Kingdom.; ^10^ Department of Applied Sciences, Northumbria University, Newcastle, United Kingdom.; ^11^ Faculty of Engineering, Department of Earth Science & Engineering, Imperial College, London, United Kingdom.; ^12^ Independent Consultant for the US National Academies of Sciences (NAS), Washington, District of Columbia.; ^13^ Carl Sagan Center, SETI, Mountain View, California.; ^14^ Institut für Experimentelle und Angewandte Physik, Abteilung Extraterrestrische Physik, Christian-Albrechts-Universität zu Kiel, Kiel, Germany.; ^15^ Eurospace, Paris, France.

**Keywords:** Icy moons, Europa, Enceladus, Planetary protection, Requirements, Spacecraft

## Abstract

To ensure that scientific investments in space exploration are not compromised by terrestrial contamination of celestial bodies, special care needs to be taken to preserve planetary conditions for future astrobiological exploration. Significant effort has been made and is being taken to address planetary protection in the context of inner Solar System exploration. In particular for missions to Mars, detailed internationally accepted guidelines have been established. For missions to the icy moons in the outer Solar System, Europa and Enceladus, the planetary protection requirements are so far based on a probabilistic approach and a conservative estimate of poorly known parameters. One objective of the European Commission-funded project, Planetary Protection of Outer Solar System, was to assess the existing planetary protection approach, to identify inherent knowledge gaps, and to recommend scientific investigations necessary to update the requirements for missions to the icy moons.

## 1. Preface

Planetary protection is aimed to control contamination between Earth and other bodies in the context of space exploration missions. To ensure that scientific investment in space exploration is not compromised by terrestrial contamination, special care needs to be taken by all actors and stakeholders. Committee on Space Research (COSPAR)'s current recommendations are mainly focused on requirements for space missions to Mars. The requirements for missions to the outer Solar System are currently based on quantitative probability of contamination constraints—similar to requirements applicable for missions to Mars several decades ago before easier to implement quantitative bioburden control requirements have been issued.

With increasing evidence for the presence of liquid water in the outer Solar System, the accepted number of potentially habitable environments has increased, and, in consequence, the issue of contaminating other moons and planets is becoming more important and relevant. There are several ongoing missions to planets and small bodies beyond Mars. Space agencies are currently planning missions of astrobiological interest to promising targets such as Ganymede, Enceladus, and Europa. Therefore, updating the COSPAR Planetary Protection Policy is timely and of the utmost importance.

In March 2016, the project “Planetary Protection of the Outer Solar System” (PPOSS) funded by the European Commission's H2020 Programme was kicked off. This project was intended to provide an international platform and forum where various communities (*e.g.*, policy makers, scientific community, and industry) gather and exchange views on the matter of planetary protection with a focus on outer Solar System bodies. Through an intensive 3-year program (2016–2018), the project has nurtured and catalyzed discussions to define strategic science and policy recommendations related to the prevention of biological and organic contamination in the frame of the exploration of the outer Solar System bodies.

The identification of scientific challenges and the assessment of scientific planetary protection requirements are the major focus of the PPOSS project. For this purpose, the PPOSS consortium (Appendix 1) gathered two study groups of international and multidisciplinary experts (see Appendix 2 for the composition of the study groups) to evaluate the current COSPAR Planetary Protection Policy and associated requirements, and to provide new insights and advice. Two different workshops were organized between January and April 2017 in Cologne and Florence, respectively. The discussions and outcome of these workshops as well as additional information, comments, and suggestions obtained from further experts through e-mail exchange and during the 42nd COSPAR Assembly in Pasadena, CA, USA in July 2018 were used as direct inputs into the formulation of the recommendations contained in the present report.

## 2. Introduction to Planetary Protection

From the dawn of space exploration, the international scientific community raised concerns regarding potential lunar and planetary contamination. Indeed, the need to protect celestial bodies was among the earliest space-related policies to be drafted (Tennen, 2004). Planetary protection is an international endeavor to preserve the capability to study planets and moons of astrobiological interest as they exist in their natural state. The contamination with Earth organisms and organic molecules that could be misinterpreted as biosignatures would compromise life detection missions (forward contamination). Moreover, Earth's biosphere needs to be protected from potential harmful extraterrestrial matter carried by a spacecraft returning from an interplanetary mission (backward contamination).

Maintaining and promulgating the COSPAR planetary protection policy and the associated requirements at an international level are under the mandate of COSPAR. In the past, COSPAR's Planetary Protection Policy was mainly focused on planets close to the Sun, in particular Mars, and on their protection from biological contamination. Although the requirements for Mars and the guidelines as to how these may be implemented are based on decades of experience, the necessary measures to study chemical evolution and the potential origin of life in the outer bodies of the Solar System, particularly at Europa and Enceladus, are less developed. Current guidelines are based on conservative estimates of poorly known parameters (Kminek *et al.*, 2017) and given the increased number of planned missions to ever more distant planetary bodies, an updated policy and easier to implement requirements are needed.

To date, the COSPAR Planetary Protection Policy has identified five categories of planetary protection requirements depending on the type of mission, the target body, and the types of scientific investigations involved. For category I missions, no planetary protection measures are necessary, but for category II to V missions, the planetary protection requirements become increasingly stringent depending on the scientific focus of a particular mission and on the astrobiological relevance of their individual mission target. The COSPAR Planetary Protection Policy lists category specifications for individual target bodies and mission types and assigns different categories to individual icy moon and other outer Solar System bodies in the appendix (Kminek *et al.*, 2017). A short summary is given in Table 1.

**Table 1.** Planetary Protection Categories for Icy Moons and Other Solar System Bodies

*Category*	*Mission type*	*Target*
I	Flyby, orbiter, lander	Undifferentiated metamorphosed asteroids; Io; others TBD
II	Flyby, orbiter, lander	Comets; Carbonaceous Chondrite Asteroids; Jupiter; Saturn; Uranus; Neptune; Ganymede^a^; Callisto; Titan^a^; Triton^a^; Pluto/Charon^a^; Ceres; Kuiper Belt Objects >1/2 the size of Pluto^a^; Kuiper Belt Objects <1/2 the size of Pluto; others TBD
III	Flyby, orbiters	Europa; Enceladus; others TBD
IV	Lander missions	Europa; Enceladus; others TBD
V	Restricted Earth return	Europa; others TBD

^a^ The mission-specific assignment of these bodies to Category II must be supported by an analysis of the “remote” potential for contamination of the liquid water environments that may exist beneath their surfaces (a probability of introducing a single viable terrestrial organism of <1 × 10^−4^), addressing both the existence of such environments and the prospects of accessing them.

TBD, to be determined.

### 2.1. Planetary protection requirements for Europa and Enceladus

In addition to the general assignment of target mission types to planetary protection categories, the COSPAR Planetary Protection policy states in its appendix “that requirements for Europa and Enceladus flybys, orbiters and landers, including bioburden reduction, shall be applied in order to reduce the probability of inadvertent contamination of an europan ocean to less than 1 × 10^−4^ per mission.” These requirements, based on the Coleman–Sagan formulation of contamination risk, will be refined in future years, but the calculation of this probability should include a conservative estimate of poorly known parameters, and address the following factors, at a minimum:

- Bioburden at launch
- Cruise survival for contaminating organisms
- Organism survival in the radiation environment adjacent to Europa or Enceladus
- Probability of landing on Europa or Enceladus
- The mechanisms and timescales of transport to the europan or enceladian subsurface liquid water environment
- Organism survival and proliferation before, during, and after subsurface transfer.

Missions to Enceladus are not explicitly mentioned here, but the same requirements can be assumed to be relevant for Enceladus.

### 2.2. Planetary protection requirements for small Solar System bodies

Small Solar System objects are a very heterogeneous class of objects. Therefore, general planetary protection requirements for those that are not discussed elsewhere in the COSPAR Planetary Protection Policy cannot be formulated but have to be addressed on a case-by-case basis to assign a planetary protection mission category. For Earth return missions, the decision between category V restricted or unrestricted Earth return has to be based on the answers to the six questions formulated in the policy (Table 2). For containment procedures to be required (“Restricted Earth return”), an answer of “no” or “uncertain” needs to be returned to all six questions.

**Table 2.** COSPAR Planetary Protection Policy Questions for the Categorization of Sample Return Missions from Small Solar System Bodies

1	Does the preponderance of scientific evidence indicate that there was never liquid water in or on the target body?
2	Does the preponderance of scientific evidence indicate that metabolically useful energy sources were never present?
3	Does the preponderance of scientific evidence indicate that there was never sufficient organic matter (or CO_2_ or carbonates and an appropriate source of reducing equivalents) in or on the target body to support life?
4	Does the preponderance of scientific evidence indicate that subsequent to the disappearance of liquid water, the target body has been subjected to extreme temperatures (*i.e.*, >160°C)?
5	Does the preponderance of scientific evidence indicate that there is or was sufficient radiation for biological sterilization of terrestrial life forms?
6	Does the preponderance of scientific evidence indicate that there has been a natural influx to Earth, for example, through meteorites, of material equivalent to a sample returned from the target body?

COSPAR, Committee on Space Research.

### 2.3. Knowledge gaps

Our knowledge about the environmental conditions, chemical composition, and geological and mineralogical processes of the mission targets in the outer Solar System is often very limited. Some of the information necessary for the COSPAR Planetary Protection implementation measures for icy moons and other outer Solar System bodies can only be obtained by future space missions and research projects. The duration of such research activities as well as the associated issues of planning uncertainties in terms of schedule, cost, and acceptance go beyond the scope of a single space project. Mission planners and project experts need more detailed guidance on how to comply with these requirements. The implementation has to be based on traceable, testable, and measurable parameters at comparable levels of confidence.

### 2.4. Previous alternative suggestion for PPOSS bodies

An alternative approach to the current COSPAR Planetary Protection Policy was suggested by the Committee on Planetary Protection Standards for Icy Bodies in the outer Solar System in 2012 in the report “The Assessment of Planetary Requirements for Spacecraft Missions to Icy Solar System Bodies” (Sogin *et al.*, 2012). The committee did not recommend the use of the Coleman–Sagan formulation to estimate the probability of contaminating outer Solar System icy bodies, because this calculation included multiple factors of uncertain magnitude that often lack statistical independence. It suggested instead a series of seven hierarchically organized independent binary decisions that reflect the environmental conditions and geological processes on the target body in the context of metabolic and physiological diversity of terrestrial microorganisms (Table 3). If the answer to at least one of the seven questions would be “no,” the entire spacecraft must be subjected to a terminal bioburden reduction process to meet planetary protection guidelines.

**Table 3.** Suggested Binary Decision Tree for Icy Solar System Bodies from the Committee on Planetary Protection Standards for Icy Bodies in the Outer Solar System

1	Liquid water: Do current data indicate that the destination lacks liquid water essential for terrestrial life?
2	Key elements: Do current data indicate that the destination lacks any of the key elements (*i.e.*, carbon, hydrogen, nitrogen, phosphorus, sulfur, potassium, magnesium, calcium, oxygen, and iron) required for terrestrial life?
3	Physical conditions: Do current data indicate that the physical properties of the target body are incompatible with known extreme conditions for terrestrial life?
4	Chemical energy: Do current data indicate that the environment lacks an accessible source of chemical energy?
5	Contacting habitable environments: Do current data indicate that the probability of the spacecraft contacting a habitable environment within 1000 years is less than 10^−4^?
6	Complex nutrients: Do current data indicate that the lack of complex and heterogeneous organic nutrients in aqueous environments will prevent the survival of irradiated and desiccated microbes?
7	Minimal planetary protection: Do current data indicate that heat treatment of the spacecraft at 60°C for 5 h will eliminate all physiological groups that can propagate on the target body?

### 2.5. Different planetary protection challenges for Mars and icy moon missions

In the past, astrobiological exploration in our Solar System has focused mainly on the planet Mars with potential modern habitable areas to be defined as “special regions.” These special regions are areas that may accommodate conditions allowing the survival and replication of terrestrial microorganisms (Rettberg *et al.*, 2015, 2016). The delivery of terrestrial microorganisms to Mars and their subsequent survival and proliferation in these “special regions” can compromise the discovery of pristine evidence of martian life. An environment of biotic relevance, that is, a habitable niche, can have different dimensions from several kilometers to a few micrometers. Different environments, which may be important for present-day life on Mars, have already been documented. These include liquid brines in the near subsurface and impact-related hydrothermal systems, where life could exist if essential elements and an energy source are present (Rummel *et al.*, 2014).

The icy moons in our Solar System are another target of great interest for astrobiology. For some of these icy moons, there is direct evidence for the existence of an ocean underneath the surface, for others there is only indirect evidence. The Cassini mission detected a hydrothermally driven plume from Enceladus (Spencer and Nimmo, 2013; McKay *et al.*, 2014; Hsu *et al.*, 2015). Magnetic field data are best explained by highly conductive saline water below the surface of Europa (Kivelson *et al.*, 2000), Ganymede (Kivelson *et al.*, 2002), and Callisto (Kivelson *et al.*, 2000). The possibility of subsurface water on the icy moons has implications for the origin and distribution of life in the Solar System because liquid water is a key prerequisite for habitability (Sephton, 2004), although the case for an origin of life on the icy moons is under debate (Pascal, 2016).

Numerous flyby and lander missions are being planned to search for life on the moons and the potential of either contaminating indigenous biota or creating false positives in life detection experiments is enhanced by the presence of water. False positive results are generated when a potential terrestrial signal is detected that can be confused with an extraterrestrial signal from biological materials. On Mars, organic compounds are likely to be scarce and the major challenge is their detection. Nonbiological organic compounds can originate from meteoric or cometary material or through synthesis in hydrothermal systems. These organic carbon species can thereafter be transformed through chemical processes that are not very well understood (Mahaffy *et al.*, 2004). The radiation environment of Mars can promote the survival of small organic compounds (Pavlov *et al.*, 2012). On icy moons, organic compounds are likely to be plentiful and the major challenge lies in diagnosing their source. Polymerization of simple organic compounds is favored under icy moon conditions (Kimura and Kitadai, 2015). High radiation environments may produce amino acids and their oligomers, polymers, or macromolecules (Cassidy *et al.*, 2010; Neish *et al.*, 2010; He and Smith, 2014). Likewise, contaminating Earth microorganisms (dead or alive) and their radiation-induced decomposition products can be a source of false positive results in direct life detection or biosignature identification. On Mars, the low abundances of organic compounds can lead to false positives from terrestrial contamination, whereas on icy moons, the detection of polymers and macromolecules can lead to false positives when abiopolymers are confused with biopolymers and are regarded as biosignatures.

To summarize, there are significant differences between Mars and the icy moons, the former is dry and organic matter-poor, whereas the latter are potentially wet and organic matter rich. Contamination on Mars can be expected to comprise local isolated events, even if a transfer of contaminating agents can occur, for example, by dust storms, whereas contamination of subsurface oceans on icy moons can potentially become global. Therefore, planetary protection requirements and contamination prevention measures have to take this into account.

Statement 1^*^: The recent extension of astrobiological exploration of the outer Solar System from Mars to the icy moons requires reconsideration of the existing planetary protection requirements for icy moons.

## 3. Icy Moons

Moons in the outer Solar System—especially Jupiter's moon Europa and Saturn's moon Enceladus—are promising targets for finding complex organic chemistry and possibly life due to the presence of liquid water under their icy shells. The inner part of the icy moons is presumably formed by a similar rocky core of silicate or metallic rocks—however, the interior structure may vary. The assumed subsurface oceans of the icy moons require an energy source for keeping the water in the liquid state. Unlike Earth's oceans, which are heated from above by sunlight, the subsurface oceans of outer Solar System bodies would be heated from below by the rocky core, and possibly from within by tidal friction (Tyler, 2008). The ice sheet at the surface can form a stagnant or mobile lid allowing convection to a certain degree. The ocean may have a direct contact to the rocky core or to an additional high-pressure ice phase at the bottom. The latter would inhibit the utilization of minerals by life forms.

Common to these icy moons are the low temperatures at the surface and in the subsurface ocean, as well as the negligible atmospheric pressure (with the exception of Titan), resulting in surface conditions far from equilibrium with liquid water. At the surface, water in the form of ice sublimates into vacuum. In some cases, cryovolcanism, as well as geysers, has been observed. The mechanisms and extent of vertical material transport from the subsurface to the surface are controlled by gravity, heat flow, and ice thickness. Vertical transport in the ice shell can be based on top–down or bottom–up processes. Examples for the former are impact gardening, radiation damage, ion implantation, tensile surface fractures, and burial. Bottom–up process can be induced by melting, convection, or fluid-filled cracks. To date, it is not clear whether material exchange in both directions takes place or whether there is an area between the surface and the subsurface, which is not mixed at all.

The temperature of the water in contact with the base of the ice shell is limited to <0°C. Any excess energy would induce melting of the ice until a new equilibrium is reached. Heat deposition from below or within, combined with cooling from the top, leads to convective motions within the ocean that will rapidly mix and homogenize the temperature of the ocean to be in equilibrium with the ice shell (Vance and Goodman, 2009). Only if the ocean has a very low salinity and a thin ice shell, the thermal expansion minimum of water can result in the creation of a cold liquid at the top, which would allow for significant (still <4°C) excursions >0°C in the bulk ocean water (Melosh *et al.*, 2004). Experiments and models scaling hydrothermal plumes on Europa show that even with a large energy input and high water temperature at the seafloor, which once the water is more than ∼1 km from the source, it has mixed with ambient water to lower the temperature anomaly to ∼1 mK above the surrounding ocean water (Goodman *et al.*, 2004). Thus, liquid water at temperatures that deviate significantly (tens of degrees) above freezing should only exist in porous channels below the silicate seafloor, or in the immediate vicinity (approximately meters) of a hydrothermal vent at the seafloor.

### 3.1. Europa and Enceladus

Europa is the smallest of the four Galilean moons of Jupiter with a size similar to Earth's moon. It has a young dynamic water ice surface. Beneath the ice, a liquid salty ocean is assumed based on magnetometer measurements and the surface characteristics imaged by remote sensing from the Galileo spacecraft (Khurana *et al.*, 1998, 2008; Pappalardo *et al.*, 1999; Kivelson *et al.*, 2002; Greeley *et al.*, 2004). The surface temperature of Europa is ∼80 K in the polar regions and ∼120 K in the equatorial regions (Spencer *et al.*, 1999). The estimated temperature in the salty ocean is <0°C due to the reduced freezing point of water by dissolved salts. Europa's surface is covered with double ridges, whose exact formation mechanism remains unknown, but is thought to involve tidal cracking of the surface ice and heating from below. Another common feature type is bands formed by separation and spreading of the crust and upwelling of warm ice from below. A significant fraction of Europa's surface is covered by chaotic terrain, where some endogenic heat source has mobilized warm ice and/or shallow liquid water to disrupt pre-existing surface features. The chemical composition of the subsurface ocean is unknown; however, salts such as hydrates of magnesium and sodium sulfates and carbonates have been identified around tectonic features (McCord *et al.*, 2010), but their concentration and the possible presence of additional salts and other chemical compounds are not yet known (Kargel *et al.*, 2000). Thickness estimations of the ice layer are in the range of a few tens of kilometers, for example, at impact crater sites ∼15 km (Schenk and Turtle, 2009). The distance from Europa's surface to the top of the rocky core is assumed to be 80–170 km (Anderson *et al.*, 1998). Europa has a very thin atmosphere (0.1 μPa) composed of molecular oxygen. Some estimations hint at the possibility that Europa's ocean might be oxygenated (Greenberg, 2010), whereas other models predict a more reduced ocean.

Enceladus is the sixth largest moon of Saturn with a diameter of about 500 km and an average surface temperature of about 75 K with ∼33 K at the north pole and 145 K at the south pole. The surface has a high albedo and is characterized by several regions of cratered terrain, regions of smooth terrain, and lanes of ridged terrain. In addition, extensive linear cracks and scarps were observed as well as impact craters. A subsurface ocean is assumed to exist beneath the ice layer. Water-rich plumes are emitted near the south pole where the ice thickness is estimated to be ∼10 km, indicating local current endogenic geological activities. The ice shell in the equatorial region was modeled to be >30 km (Hsu *et al.*, 2015). The distance from the surface of Enceladus to the top of the rocky core is estimated to be 60 km (Iess *et al.*, 2014). The cryovolcanoes expel geyser-like jets of water vapor and solid material, including sodium chloride crystals, silica and ice particles, carbon dioxide, molecular hydrogen, methane, ammonium, and small quantities of heavier hydrocarbons and other organic molecules (Spencer and Nimmo, 2013; Waite *et al.*, 2017) into space.

### 3.2. The radiation environment in the outer Solar System

The space radiation environment in the outer Solar System is composed of solar particles, mainly electrons, protons, and alpha particles, as well as galactic cosmic rays, which consist nearly exclusively of ions from protons up to uranium. Solar particle events cannot be forecasted, but have the highest probability around solar activity maxima. The Mars Science Laboratory Radiation Assessment Detector (RAD) instrument (Hassler *et al.*, 2012) actively measured the dose rate en route to Mars and found a mean value of 77 micro-Sieverts per hour (Zeitlin *et al.*, 2013). Because of the heliospheric modulation of the predominantly galactic cosmic radiation, this is expected to increase slightly, by 4.5% ± 0.5% per astronomical units (AU) on the way to the outer planets (Gieseler *et al.*, 2008). The decline in solar activity in the past 3 years has resulted in an increase of the galactic cosmic radiation flux of ∼30% at the Moon (Schwadron *et al.*, 2018). This illustrates the substantial variability of the background radiation field en route to the outer planets. On the surface of Mars, RAD measured a dose rate of 26 micro-Sieverts per hour, but this too has increased by ∼50% in the past 3 years.

Some information is available inside the jovian system, it has been summarized in Paranicas *et al.* (2007, 2009). The most extreme particle radiation fluxes are seen in the orbital regimes between Io and Europa (Cooper *et al.*, 2001; Jun *et al.*, 2005; Paranicas *et al.*, 2008). This radiation is less intense at Enceladus' orbit around Saturn (Garret *et al.*, 2005). The magnetospheres of Jupiter and Saturn also exhibit high fluxes of energetic electrons that can interact with the surface layers of Europa and Enceladus down to a depth of the order of a meter or so if one also considers their bremsstrahlung photons. Heavy ions induce severe, and difficult to repair, damages in all living organisms (Cucinotta *et al.*, 2017; Moeller *et al.*, 2017). Data regarding the radiation environment on and around Europa from the Voyager and Galileo spacecraft were used to model the depth profile of radiation within the europan ice (Paranicas *et al.*, 2007, 2009). The penetration depth depends on the type and energy of the particle. The derived yearly radiation dose at 1 m depth was calculated to be 0.3 Gy/a. Thus, only a few hundred Gray would be accumulated during a stay in that depth of 1000 years, if no transfer or exchange with the deeper subsurface occurs. As described in the next paragraph, this radiation dose can be tolerated by many Earth microorganisms.

For the radiation environment of Enceladus, less data are available. However, lower radiation dose rates are expected within the orbit of Enceladus than Europa because the energy and flux of the saturnian magnetospheric electrons are significantly lower than those of jovian magnetospheric electrons.

In addition to ionizing radiation, solar UV radiation is also an important factor in the outer Solar System. The distance between the jovian system and the Sun is 5.2 AU (the distance from Earth to the Sun), leading to a calculated UV irradiance of ∼3.7% of that in low Earth orbit. Although life on Earth is protected by the ozone layer from UV wavelengths shorter than 295 nm, the extremely thin or nonexisting atmospheres of the icy moons are no barrier for those wavelengths. In space and on any celestial body without a significantly UV-absorbing atmosphere, this short wavelength UV radiation reaches the surface. On Mars, wavelengths as low as 200 nm are transmitted through the CO_2_ dominated atmosphere of 600–1000 Pa pressure. Owing to their low gravity, the icy moons cannot hold UV filtering atmospheres: Europa's atmosphere is mainly constituted of O_2_ with pressures <10^−6^ Pa, similar to those of Ganymede and Callisto; Enceladus' trace atmosphere consists mainly of water vapor. Thus, without atmospheric attenuation, the jovian and saturnian systems, which are 5.2 and 9.6 times farther away from the Sun than Earth, experience an irradiance of 3.7% and 1.1% of that measured outside Earth's ozone layer.

## 4. Life As We Know It

Even if there is no generally accepted unambiguous definition of life, all life forms on Earth, the only inhabited planet we know, are characterized by certain properties. Organisms are composed of cells, maintain homeostasis, and perform metabolic reactions by exploiting thermodynamic disequilibria for gaining energy from redox reactions. They use compounds from the environment for building up their biomass. They are able to respond to stimuli, adapt to changing environmental conditions, and reproduce and undergo Darwinian evolution. Furthermore, all organisms depend on water as a solvent, for stabilizing the structure of complex molecules and as a partner in biochemical reactions.

Prokaryotic and eukaryotic microorganisms dominate life on Earth with respect to the number of individuals, diversity, and biomass. They are ubiquitous and present in almost all places on Earth, even in “extreme environments,” for example, permafrost, glacier ice, hot and cold deserts, salt evaporates, hydrothermal vents, the deep subsurface, hot springs, deep-sea brines, and alkaline and acidic lakes. Microorganisms living in such environments are also known as extremophiles, whereas some are even polyextremophilic, that is, adapted to two or more extreme parameters (Harrison *et al.*, 2013). One example is microorganisms living in deep-sea brines where they are exposed to very high salinity (low water activity), high pressure, increased temperature, and high concentrations of heavy metals (Antunes *et al.* 2011, 2015).

Such environments are called extreme from a human point of view but might not be extreme for the organisms living there. Indeed, such microorganisms do not only survive in such extreme conditions but also require these conditions for metabolism and replication. However, it should be kept in mind that organisms are adapted to their specific habitat, that is, organisms thriving at one end of the parameter range will not grow at the other end. For example, organisms living at very high salt concentrations will not grow at low salt concentrations and those living at high pH values are not able to live at low pH values. Our knowledge about the physiological responses of extremophiles to multiple stressors and their ability to replicate under these conditions is very limited (Harrison *et al.*, 2013). It must be noted that in a dormant or anhydrobiosis state, organisms can survive in more “extreme” environments than active metabolizing organisms, which are able to replicate. The ranges for the former are much broader than the latter (Table 4).

**Table 4.** Limits of Growth and Survival (Modified from Moissl-Eichinger *et al.*, 2016)

*Environment*	*Limits of growth*	*Limits of survival*
Temperature	−25°C to +122°C	−263°C to +122°C
Water stress	*a*_w_ ≥ 0.61	0 ≤ *a*_w_ ≤ 1.0
Low pressure	0.7 kPa	For example, spores survive vacuum (10^−6^ Pa)
Salinity	Up to saturation	Salt crystals (endoevaporites)
pH	pH = 0–11.5	pH = 0–12.5
Nutrients	High metabolic versatility	Not required, better without
	Lithoautotrophic growth	
	High starvation tolerance	
Oxygen	Anaerobic growth	Not required, better without
	Aerobic growth, facultative to obligate	
Radiation	High UV sensitivity, radiation sensitivity (<6 kGy, <60 Gy/h)	Reduced UV and radiation sensitivity at low temperatures
Pressure	100 MPa (138 MPa in NaCl)	
Time	Hours to months	≤25–40 × 10^6^ years

w, water activity.

**Statement 2:**

(a) Microorganisms are ubiquitous and can live in extreme environments.
(b) Organisms are adapted to their specific habitat.
(c) The limits for survival are broader than for replication.

The availability of liquid water is central for life, allowing it to persist and to multiply. Microorganisms that prevail in environments with low water activity are either xerophilic organisms, which have been mostly isolated from preserved food and arid environments, or halophilic, isolated from natural or artificially created hypersaline sites, where water availability is usually limited by high NaCl concentrations. Xerophiles isolated from high sugar content food are often eukaryotes. Some filamentous fungi can still germinate at values as low as 0.650–0.605 *a*_w_ (Stevenson *et al.*, 2015) with calculations further reducing the lower limits to 0.632 and 0.636 *a*_w_ for *Aspergillus penicilloides* and *Xeromyces bisporus*, respectively. Salty environments are usually dominated by prokaryotes, particularly members of the archaea. *Haloarchaea* are found in environments with NaCl levels up to saturation and a resulting *a*_w_ of 0.75 and were found to survive inside salt crystals or in ancient salt deposits for long time periods (Grant, 2004; Stevenson *et al.*, 2015). Halophilic microorganisms have been shown to grow <0.7 *a*_w_, for example, *Halococcus salifodinae* (0.693), *Halobacterium noricense* (0.687), *Natrinema pallidum* (0.681), and haloarchaeal strains GN-2 and GN-5 (0.635 *a*_w_). Theoretical lower limits of 0.611 *a*_w_ have been calculated for extremely halophilic archaea and bacteria based on extrapolated growth curves. Collectively, these findings suggest that there is a common water-activity limit that is determined by physicochemical constraints. To cope with the high osmotic stress imposed on the organisms in the low water activity environments, a minority of halophiles use counterbalancing levels of inorganic ions (usually KCl); whereas the majority of halophiles, in particular halophilic archaea, and all xerophiles produce or accumulate low-molecular mass organic compounds with osmotic potential (compatible solutes such as trehalose) to achieve osmotic stability.

Vacuum as experienced in space is devoid of any available water. Low pressure of 10^−3^ to 10^−7^ Pa renders active life and metabolism impossible. Nevertheless, a long-term space mission proved that, in an inactive anhydrous state, some microorganisms can survive up to 6 years, and probably longer. In particular, spore formers like bacilli are resistant against the low-pressure vacuum, but also vegetative cells of some species adapted to environments that repeatedly dried in their natural environment can survive space vacuum exposure on space missions or simulated in the laboratory for several months (Table 5) (Billi *et al.*, 2011; Bryce *et al.*, 2015).

**Table 5.** Examples for Bacterial Survival in Space Vacuum

*Mission*	*Organism*	*Duration of exposure (days)*	*Pressure (Pa)*	*% survival in thin layers*	*References*
LDEF	*Bacillus subtilis*	2107	*10^−6^*	1.4 ± 0.8	Horneck (1993); Horneck *et al.* (1994a)
EURECA	*B. subtilis*	327	*10^−5^*	32.1 ± 16.3	Horneck *et al.* (1995)
EXPOSE-R2	*Deinococcus geothermalis*	672	*10^−3^ to 10^−4^*	0.1 ± 0.01	Rabbow *et al.* (2017); Panitz *et al.* (in press)

EURECA, European Retrievable Carriere; LDEF, Long Duration Exposure Facility.

In contrast, barophiles or piezophiles are able to survive high pressures. Barophilic organisms have been isolated from deep subsurface areas or oceans. For example, in the laboratory, *Shewanella oneidensis* remained metabolically active at up to 1000 MPa (Sharma *et al.*, 2002). Interestingly, at that pressure, liquid water should not be available at any temperature.

For microbial life to exist, it also needs an energy source. On Earth, primary production is driven by photosynthesis where energy is gained from the Sun; however, in subsurface environments, where there is no sunlight, primary production is driven by chemotrophs, which gain energy from chemical compounds (Madigan *et al.*, 2009; Oremland *et al.*, 2017). Chemotrophs can harvest energy from reduction–oxidation (redox) reactions using inorganic (lithotrophs) or organic (organotrophs) substrates that are available in the environment. For a review about bacterial redox sensors, please refer to Green and Paget (2004) and references therein. Microorganisms also need a carbon source and some organisms can fix carbon dioxide (called autotrophs); whereas others need an organic carbon source (heterotroph) (Cockell *et al.*, 2016). Virtually all trace organic compounds can support heterotrophic life. Regarding the icy moons, chemoautotrophs are most important, because they can utilize either organic or inorganic compounds, such as iron, hydrogen, or sulfur for metabolism, which are potentially present in the subsurface oceans.

On Earth, the magnetic field effectively shields life from galactic cosmic radiation and heavy ions. In space, and on the icy moon's surface (Paranicas *et al.*, 2007, 2009), microorganisms will be exposed to this high-energy type radiation. Galactic cosmic radiation and HZE (from high [H] atomic number [Z] and energy [E]) particles cannot be fully shielded by a spacecraft during a long-term space travel. However, a column of water or ice, as expected on the icy moons, reduces the dose effectively to a calculated radiation dose at 1 m depth of 0.3 Gy/a for the Europa moon, providing a possible habitat in the radiation shielded subsurface ocean (Paranicas *et al.*, 2007, 2009). Although radiation on one side is a driver for evolution by mutation induction, on the other side, ionizing radiation is the most deleterious environmental parameter and highly inactivating for microorganisms. The calculated Europa dose rate for ionizing radiation is harmful for most of the higher eukaryotic organisms. A very few radiation-induced DNA double strand breaks per chromosome lead to death in most organisms (Resnick, 1978). Yet, either by evolving cellular mechanisms that protect the proteome and DNA from radiation-induced damage, or by developing highly efficient DNA repair mechanisms, some organisms achieve a high radiation resistance (Pavlopoulou *et al.*, 2016).

Examples of very radiation-resistant organisms are the mesophilic bacterium *Deinococcus radiodurans*, withstanding radiation doses of up to 5000 Gy without loss of viability (Moseley, 1983). Also hyperthermophilic archaea such as *Thermococcus stetteri*, and bacteria such as *Ignicoccus hospitalis* and *Aquifex pyrophilus* (Beblo *et al.*, 2011) can tolerate radiation doses up to several thousand Gray comparable with the dose at 1 m depth on Europa accumulated in about 20,000 years, that is, if no transfer and exchange with the deeper subsurface occur. For Enceladus, less data are available. However, lower radiation dose rates are expected within the orbit of Enceladus at Saturn than Europa. Therefore, the chance of microorganisms surviving on the upper layer of the surface ice of Enceladus is even higher than on Europa. Interestingly, irradiation performed at low temperatures of −79°C even increased the resistance of *D. radiodurans* (Dartnell *et al.*, 2010). Nevertheless, less radiation-resistant microorganisms, for example, *Bacillus subtilis*, were exposed to galactic cosmic radiation and HZE particles on the long-term missions Exobiology Radiation Assembly on the European Retrievable Carriere (EURECA) and the Long Duration Exposure Facility (LDEF). On EURECA, 25% of the *B. subtilis* spores exposed to space radiation of up to 0.41 Gy for 327 days in vacuum survived (Horneck *et al.*, 1995), whereas on LDEF, still 1% to 2% of spores remained viable after a total galactic cosmic radiation of 4.8 Gy experienced in the 2107 day space mission (Horneck *et al.*, 1994a).

The effect of HZE particles on life was investigated on ground utilizing heavy ion accelerators and a variety of microorganisms. Cross sections of the investigated endpoints exhibited a similar dependence on energy as measured in space experiments. For light ions with *Z* ≤ 4, they decreased with increasing energy, becoming independent from energy for *Z* around 10 and finally increasing with energy for ions with *Z* ≥ 26 (Baltschukat and Horneck, 1991; Horneck *et al.*, 1994). DNA double strand breaks induced by heavy ions are the most severe DNA damage, leading in most organisms to a disintegration of the DNA that cannot or only insufficiently be repaired. About 100 double strand breaks per chromosome are induced by irradiation with 10,000 Gy in the mentioned *D. radiodurans* as shown by pulsed-field gel electrophoresis and successfully repaired within 29 h without any loss of viability (Daly and Minton, 1996). In a recent series of experiments, different organisms from bacteria, archaea, fungi, lichens, to rotifers were exposed to very high doses of X-rays and HZE particles. The analysis of various biological endpoints with a combination of different biochemical and molecular biological methods, for example, vitality, survivability, cell proliferation, and damage induction, showed a remarkable radiation resistance in many organisms (Moeller *et al.*, 2017, and the references cited therein).

The present-day atmosphere of Earth contains about 21% oxygen and a stratospheric ozone layer that absorbs the solar UV radiation with wavelengths <290 nm. However, on the early Earth, when life evolved, the UV climate was different. The atmosphere was anoxic and energy-rich deleterious short wavelength UV radiation could penetrate the atmosphere due to the lack of an ozone layer. In contrast to the penetrating ionizing radiation, UV radiation is only harmful for microorganisms that are not shaded, hence protected, by spacecraft or planetary material or even other microorganisms. Interestingly, the survival of the radiation-resistant *D. radiodurans* is only reduced to 10% (*F*_10_) after exposure in suspension to 660 Jm^−2^ UVC radiation of 254 nm and after exposure in a dry form to 1370 Jm^−2^ (Bauermeister *et al.*, 2011). In a similar way, a range of thermophilic microorganisms survived up to 5000 Jm^−2^ monochromatic UVC of 254 nm (Beblo *et al.*, 2011). The effectiveness of the short UV range >280 nm and extraterrestrial UV in killing resistant *B. subtilis* spores was impressively demonstrated in a space experiment performed on Spacelab D2, where the reduction and loss of the Earth ozone layer were simulated and the biological effectiveness of the resulting solar short UV ranges was determined. Although the measured irradiance increased only slightly, biological effectiveness increased by a factor 1000 (Horneck *et al.*, 1996). An extensive list of ionizing and UV radiation-resistant organisms was reviewed in Gabani and Singh (2013).

Combinations of environmental parameters may act as multiple stressors, adding up to an increase of the total effect. The survivability of high radiation and desiccation resistance bacteria has been attributed not only to effective enzymatic DNA repair mechanisms but also to the protection of proteins from oxidative damage induced by desiccation (Fredrickson *et al.*, 2008; Zhai *et al.*, 2008).

## 5. Parameters Influencing the Survival of Earth Organisms with the Potential to Contaminate Icy Moons

For the planetary protection of icy moons, the only relevant organisms are those with the potential to contaminate the subsurface oceans and replicate (“problematic species”) (Box 6). This requires that Earth microorganisms are able to survive different steps over a long period of time under challenging conditions. First, the microorganisms have to enter the clean rooms used for assembly, integration, and testing of space hardware. These are microorganisms that are either associated with humans, originating from the facilities' environment, or brought in with hardware and ground support equipment. For microorganisms, spacecraft assembly clean rooms represent an extreme oligotrophic environment with constant moderate temperature, controlled air circulation, and relatively low and constant humidity. Clean rooms are subjected to strict cleaning regimes with antimicrobial and sporocidal agents (such as ECSS-Q-ST-70-01C; Venkateswaran *et al.*, 2001). Therefore, the replication of microorganisms in clean rooms is not very likely and might be limited to few localized areas within the clean room and to few species of microorganisms, if at all. Only desiccation-resistant vegetative microorganisms or spores, that is, dormant resistant forms of some bacteria, can survive there for a longer period of time.

Clean rooms used for assembly, integration, and testing of spacecraft with planetary protection requirements are biologically monitored on a regular basis. The bioburden and biodiversity in clean rooms are determined following standard procedures and assays (ECSS-Q-ST-70-55C, 2008), in addition to the obligatory bioburden measurements of the spacecraft hardware itself. After rigorous cleaning and bioburden reduction steps, the few microorganisms that remain on the spacecraft hardware alive in a dried form have to survive the transport to the launch site, the integration in the launch vehicle, the launch itself, and the long-term space travel to the final destination in the outer Solar System.

Space travel will take years depending on the mission target and the specific mission schedule. For example, the Juice mission is planned to launch in June 2022 and to arrive in the Jupiter system after 7.6 years in January 2030 (ESA, http://sci.esa.int/juice/50074-scenario-operations/), whereas Juno traveled in only 4.9 years from Earth to Jupiter (NASA, https://www.nasa.gov/mission_pages/juno/overview/index.html). In space, the combination of space vacuum, ionizing radiation, extraterrestrial solar UV radiation on the outer surfaces of the spacecraft, and low temperatures pose stringent limits on the survival of terrestrial microorganisms.

**FIG. 1.**
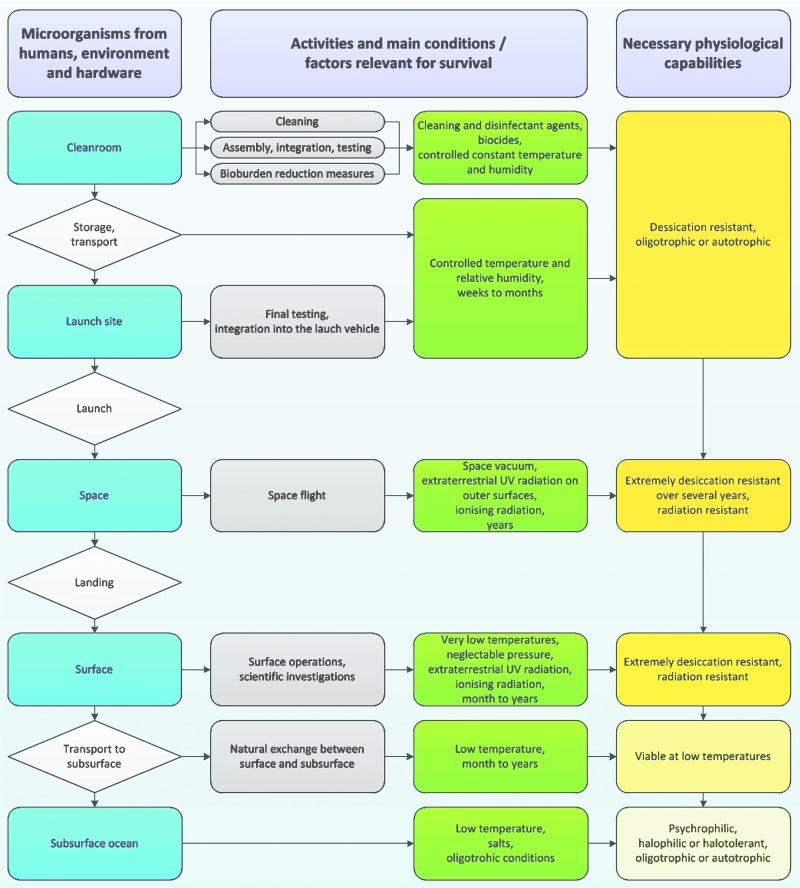
Microbial characteristics of relevant microorganisms for future missions to the outer Solar System.

**FIG. 2.**
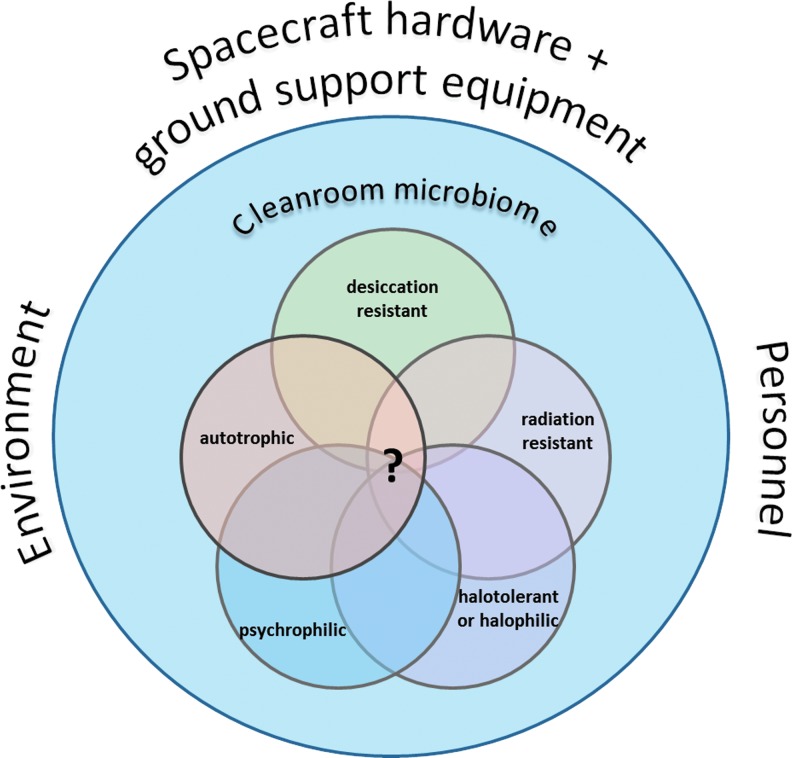
Relevant organisms with the potential to contaminate icy moons (“problematic species”) as a so far unknown subpopulation of the overall clean room microbiome.

Whereas most microorganisms can survive low temperatures in a metabolically inactive state, the extremely desiccating conditions induced by exposure to space vacuum inactivate many microorganisms, but not all, for example, spores can survive. After landing, the small percentage of microorganisms that survived until that stage also have to survive on the surface and near surface (*i.e.*, cracks) of the icy moon before being transported to the subsurface ocean. The extent, mechanism, and timescale of the exchange of materials and the potential transport of contaminating organisms between the deeper surface and the subsurface ocean are unknown. Yet, it can be assumed that contaminating organisms would survive for an extended period of time on the very cold surface. If microorganisms were able to penetrate the icy shell and enter the subsurface oceans, the temperatures will be low, that is, <0°C, so replication would be limited to psychrophilic/psychrotolerant microorganisms. It is also unknown whether dissolved minerals and other chemical compounds would be available for life. As a consequence, microorganisms are only relevant for icy moons if they are able to survive all of these challenging conditions—from their introduction into the spacecraft assembly clean room to the subsurface ocean of icy moons over several years—and are able to replicate in the subsurface ocean (Figs. 1 and 2). From laboratory experiments and field studies, we can derive information about the resistance of organisms to single and multiple icy moon relevant environmental factors, but there are also additional aspects that can influence whether or not an organism can establish itself, grow, and affect the ecology of the environment in which it arrives, in the subsurface ocean of an icy moon. Indeed, in some cases, other organisms or specific conditions might even be a prerequisite. However, these factors are not yet known or understood in sufficient detail.

**Statement 3:** Relevant organisms with the potential to contaminate icy moons are those that are able to survive long periods (years) of desiccation and vacuum, to survive long periods (years) of exposure to cosmic radiation (during cruise and on the surface of the moons), and to replicate at low temperatures under oligotrophic anoxic conditions in the presence of salts (inside or beneath the ice).

## 6. The Present-Day Planetary Protection Approach for Bioburden Determination

**FIG. 3.**
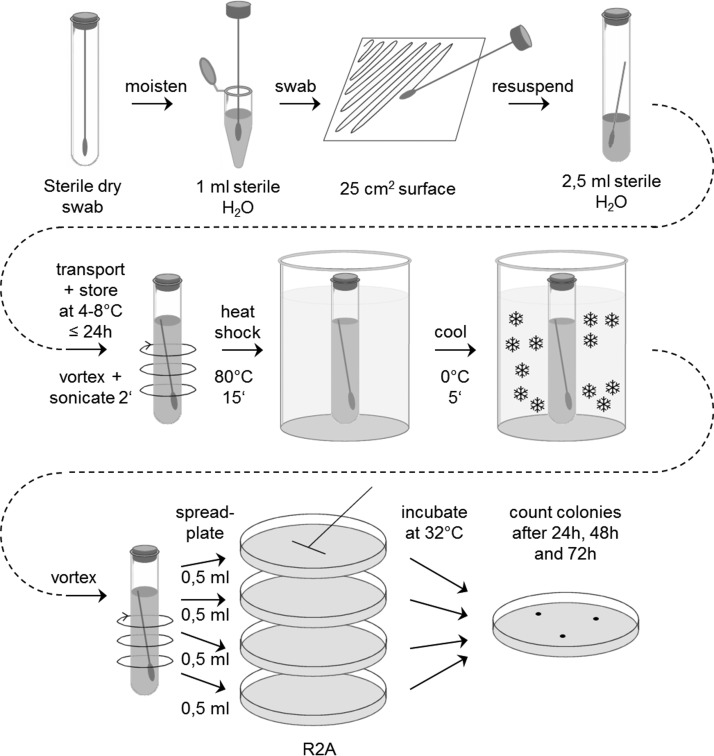
The ESA bioburden standard assay for swabs (ECSS-Q-ST-70-55C).

The bioburden limits specified in the COSPAR requirements for different mission type/target combinations are based on results from standardized cultivation assays. In two comparable standards, the detailed procedures for sampling and microbiological analysis of space hardware are described (ECSS-Q-ST-70-55C, 2008; NASA-HDBK-6022, 2010). The sampling technique employed both sterile swabs and wipes moistened with distilled water. Swabs are used for sampling small surfaces up to 25 cm^2^. Wipes are used preferentially on larger areas up to 1 m^2^. The microbiological assay procedure features the sonication of the sample with sterile distilled water or rinse solution, followed by an aerobic incubation on nutrient agar (Tryptic Soy agar [TSA] or Reasoner's 2A agar [R2A]) for 3 days at 32°C. Spore assays are performed by exposing the sample to 80°C for 15 min (Pasteurization step), followed by plating on TSA or R2A agar plates and incubation as already described. The number of colonies (colony-forming units [CFUs]) visible with the naked eye after incubation is used to calculate the overall bioburden of the hardware. An exemplary scheme of a standard planetary protection swab assay is shown in Fig. 3 (ECSS-Q-ST-70-55C). Wipe assays are performed in an analogous way.

With this assay, only mesophilic aerobic spores and bacteria that are able to survive a heat treatment for 15 min at 80°C and that can grow on the specified nutrient medium and conditions are determined. Most of them belong to the genus *Bacillus* and *Paenibacillus*. However, also *Micrococcus*, *Staphylococcus*, *Rothia*, *Acinetobacter*, *Stenotrophomonas*, and *Paracoccus* were among the heat-shock-surviving nonspore-forming bacteria (Moissl-Eichinger *et al.*, 2016). The number of microorganisms obtained with this standard assay is used as a proxy for the total number of all microorganisms present in a sample because the majority of them cannot multiply under these conditions. This standard assay is easy to perform, does not need special laboratory equipment, and the results are available after 72 h of incubation. However, the subset of all organisms that are problematic for PPOSS are not detected by this standard assay.

**Statement 4:** The ESA/NASA standard assays for bioburden determination do not identify and quantify problematic species.

## 7. The Spacecraft Assembly Clean Room Microbiome

The investigation of the microorganisms in clean rooms and on spacecraft started in the 1960s (for a summary of the history of planetary protection, see Meltzer, 2011). One of the first studies on the microbiome associated with spacecraft hardware was conducted by Puleo *et al.* (1970a), in which the microbial contamination associated with the Apollo 6 spacecraft was analyzed. In this study, 366 isolates were found and the majority of organisms recovered were indigenous to humans. Since then, many studies focused on the microbiome of clean rooms and the results are discussed hereunder.

Before the rise of molecular techniques, clean rooms and space hardware were routinely investigated by culture-dependent methods already described. These methods were employed to calculate the bioburden and investigate the diversity of the Apollo spacecrafts (Puleo *et al.*, 1970b, 1973), the Viking missions (Puelo *et al.*, 1977), and are still the standard techniques to investigate the cultivable microbiome of a clean room or space hardware (Rettberg *et al.*, 2006; NASA-HDBK-6022, 2010). The drawback of this method is that different organisms have different growth requirements (*e.g.*, different temperatures, pH, or nutrient composition) and only a small fraction can be recovered by this method (La Duc *et al.*, 2004a; Stewart, 2012). Another possibility to detect viable bacteria is the use of an adenosine triphosphate (ATP)-based detection system and was first tried in a clean room environment by Venkateswaran *et al.* (2003a). Because ATP is used by all living organisms, it can be used as an indicator for the presence of living organisms as previously described (Chappelle and Levin, 1968). Although this is a very straightforward assay, the amount of intracellular ATP varies with cell size, species, and physiological state (Venkateswaran *et al.*, 2003a), making it difficult to quantify and compare the results with other techniques such as the CFU detection method. This method has been shown to work well with clean room-associated organisms such as *Bacillus* sp. and previous research also suggests that this method is suitable to distinguish between viable and nonviable spores (Rawsthorne *et al.*, 2009; Mohapatra and La Duc, 2012). The same holds true for psychrophilic (Dieser *et al.*, 2010), heat-tolerant, and alkaliphilic organisms (Guo and Zhang, 2014).

With the increase in molecular techniques, researchers were able to elucidate the microbiome of a clean room with greater detail. The first study was presented by La Duc *et al.* (2003) wherein they describe the microbial characterization of the Mars Odyssey spacecraft and the facility used by the spacecraft by direct 16S rRNA amplification without previous culturing. Several studies have been conducted using this method (Moissl *et al.*, 2007a,b; Newcombe *et al.*, 2008; La Duc *et al.*, 2009; Vaishampayan *et al.*, 2010; Stieglmeier *et al.*, 2012), leading to the realization that these environments harbor a diverse set of microorganisms and that even archaea (Moissl *et al.*, 2008) and anaerobic organisms (Probst *et al.*, 2010) are present. Further advances such as pyrosequencing, Phylochip analysis, or next generation sequencing dramatically changed our perception of the microbiome associated with clean rooms and space hardware (La Duc *et al.*, 2012; Zhang *et al.*, 2018).

Although improved sequencing methods increased our understanding of the diversity associated with these environments, one cannot differentiate between living organisms or just the presence of the 16S rDNA signature from dead organisms. To address this issue, several studies employed propidium monoazide (PMA), a pre-PCR dye that is able to discriminate live and dead cells (Vaishampayan *et al.*, 2013a; Henrickson *et al.*, 2017). PMA is highly membrane impermeable and generally excluded from cells with an intact membrane assumed to be viable. Upon penetrating compromised cell membranes, PMA binds to DNA by intercalating and upon photoactivation, covalently binds the DNA and, therefore, making it not available for PCR amplification (Nocker *et al.*, 2006). This method has been shown to work well with clean room-associated organisms such as *Bacillus* sp. and previous research also suggests that this method is suitable to distinguish between viable and nonviable spores (Rawsthorne *et al.*, 2009; Mohapatra and La Duc, 2012). The same holds true for psychrophilic (Dieser *et al.*, 2010), heat-tolerant, and alkaliphilic organisms (Guo and Zhang, 2014). Although this method is promising, it does not work for all organisms. For example, dead or alive halophilic archaea cannot be discriminated because NaCl inhibits PMA from inhibiting PCR amplification (Barth *et al.*, 2012). Clean rooms are obviously not typical habitats for halophilic archaea, yet molecular studies have found signatures of halophilic archaea in such environments (Moissl-Eichinger, 2011), making it important to keep halophilic archaea in consideration when thinking of possible forward contamination. Fittipaldi *et al.* (2011) evaluated the viable real-time PCR using PMA method for environmental samples. The suggested approach based on the combination of three real-time PCR amplifications for each sample leads to improved viable cell estimations, but cannot exclude false positives completely.

**Recommendation 1**^**†**^**:** Investigate the applicability of methods for the discrimination of dead and alive cells from problematic species in low-biomass environments.

Several studies have characterized and described isolates from clean room environments and spacecraft, and currently there are nine newly described species and one novel genus: *Bacillus nealsonii* (Venkateswaran *et al.*, 2003b), *Bacillus odysseyi* (La Duc *et al.*, 2004b), *Paenibacillus pasadenensis*, *Paenibacillus barengoltzii* (Osman *et al.*, 2006), *Bacillus safensis* (Satomi *et al.*, 2006), *Bacillus canaveralius* (Newcombe *et al.*, 2009), *Paenibacillus purispatii* (Behrendt *et al.*, 2010), *Bacillus horneckiae* (Vaishampayan *et al.*, 2010), *Paenibacillus phoenicis* sp. nov (Benardini *et al.*, 2011), and the novel genus *Tersicoccus phoenicis* (Vaishampayan *et al.*, 2013b). Besides these properly described strains, there are several hundred strains deposited at the Deutsche Sammlung von Mikroorganismen und Zellkulturen GmbH, Braunschweig, Germany (Moissl-Eichinger *et al.*, 2012) and the ARS culture collection (Venkateswaran *et al.*, 2014) that await further characterization. Several investigations have been conducted elucidating the resistance of selected isolates against common cleaning procedures such as cleaning with 70% isopropanol, 5% hydrogen peroxide, and UV radiation, and some tested strains show increased resistance against these threats than type strains (Link *et al.*, 2004; Kempf *et al.*, 2005; Gosh *et al.*, 2010). However, not all isolates from these environments are resistant against decontamination treatment, even isolates obtained from the same clean room and the same sampling opportunity may differ in their resistance. Kempf *et al.* (2005) showed that strains of *Bacillus pumilus* that were isolated during the same sampling campaign from the Jet Propulsion Laboratory—Spacecraft Assembly Facility differ dramatically in their ability to tolerate exposure to 5% liquid H_2_O_2_ (Kempf *et al.*, 2005).

High Efficiency Particulate Air filtration, humidity and temperature control, partial overpressure (in ISO class 5 clean rooms), frequent cleaning, limited number of persons working at the same time in a clean room, and strict garment changing protocols—all these clean room maintenance procedures have strong impact on the abundance, viability, and diversity of microorganisms therein (Moissl-Eichinger *et al.*, 2015). However, with people working within these facilities, sterility cannot be accomplished. Most of the organisms found in the previously discussed studies were considered indigenous to humans such as *Staphylococcus*, *Micrococcus*, and the *Corynebacterium-Brevibacterium* group (Puleo *et al.*, 1973). The remaining isolates originate from the environment of the spacecraft assembly facility with some of them typical for the individual location and others that are generally associated with soil, dust, or plants (La Duc *et al.*, 2004a; Rettberg *et al.*, 2006; Moissl-Eichinger *et al.*, 2015). One study by Foster and Winans (1975) recovered psychrophilic microorganisms from surrounding areas where the Viking Spacecraft was assembled. Although psychrophiles were not found within the assembly area, the authors demonstrated that the employed techniques would not have detected these organisms.

The comparison of the microbiome data from different clean rooms in different countries and climate zones using the available published data is difficult, because different analysis methods have been employed. To be truly able to compare the biodiversity of spacecraft assembly facilities and space hardware, all investigations need to follow the same protocol, including the record of metadata with a clear description of the sequencing platforms, bioinformatics tools, and data bases used for analysis. Owing to rapid technological developments in this area, these protocols have to be updated on a regular basis. Reduced sequencing costs, high throughput methods, and powerful new analytical tools, which have been developed in the past few years, enable the investigation of microbial communities in great detail. However, each step from sample collection, storage, DNA extraction, sequencing, and computational and statistical methods for data analysis can induce bias and influence the interpretation of the obtained data as summarized in a recent review from Hugerth and Andersson (2017).

In addition, it is mandatory to establish and maintain an international quality controlled data repository for microbiome data from spacecraft assembly clean rooms and spacecraft. The data base format has to be flexible to allow the implementation of future optimized protocols and handle the enormously increasing data volume.

**Recommendation 2:** Define a detailed protocol for the molecular analysis of the clean room and spacecraft microbiome, including metadata with detailed information about the sequencing platforms and the bioinformatics tools used for analysis. Update this protocol on a regular basis taking new technological developments into consideration.

**Recommendation 3:** Establish and maintain an international quality-controlled microbiome data repository for data from spacecraft assembly clean rooms and spacecraft.

In all investigated spacecraft assembly clean rooms, the microbial community was found to be very diverse. Microorganisms belonging to genera with extremotolerant members were found in all clean rooms both with cultivation assays and with molecular methods (La Duc *et al.*, 2007; Moissl-Eichinger *et al.*, 2015). With only knowing the genus of a microorganism, it is not possible to predict the resistance against different environmental factors. Closely related species as well as different bacterial strains from the same species can possess a very distinct physiological potential (Kempf *et al.*, 2005; Callegan *et al.*, 2008; Faglaiarone *et al.*, 2017). So far, insights into the resistance against desiccation, radiation, low temperatures, salts, etc., and combinations of these factors cannot be deduced from the available information about the clean room microbiome.

**Statement 5:** With the present-day methods for biodiversity determination, it is not possible to identify and quantify planetary protection-relevant microorganisms for icy moons as defined in statement 3.

## 8. Bioburden Reduction by Sterilization

As already mentioned, the only microorganisms that are problematic for icy moons are those that can reach the subsurface oceans and replicate there. One aspect already addressed in “The Assessment of Planetary Requirements for Spacecraft Missions to Icy Solar System Bodies” (Sogin *et al.*, 2012) is the practice of sterilization of space hardware with dry heat. In the European Cooperation for Space Standardization (ECSS) standard ECSS-Q-ST-70-57C, different combinations of temperatures >110°C and exposure times are specified with a detailed process description. In the report from Sogin *et al.* (2012), the suggestion was made to lower the sterilization temperature under the assumption that psychrophilic or psychrotolerant organisms can be killed at lower temperatures than mesophilic organisms. The existing literature is not very exhaustive and does not support this assumption in general. Previous research supports the assumption that thermophiles generally produce more resistant spores than do mesophiles, which produce more resistant spores than do psychrophiles (Marquis and Bender, 1985). In addition, Winans *et al.* reported in 1977 that spores from psychrophilic organisms, isolated from the vicinity of the Viking spacecraft assembly area, are more susceptible to dry heat than mesophilic organisms (Winans *et al.*, 1977). Although research suggests the lower dry heat resistance of certain species, novel psychrophilic organisms have been isolated and identified, where little to nothing is known about their resistance to dry heat. In particular, the previously identified problematic species have not been investigated in this matter. Therefore, the heat resistance of problematic species should be investigated at different temperatures <110°C to find out, if the sterilization temperature for space hardware could be lowered in consequence.

**Recommendation 4:** Investigate the heat resistance at temperatures <110°C of planetary protection-relevant species for icy moons, in particular, spore formers, using standard sterilization procedures for space hardware.

## 9. Mid- and Long-Term Research Activities for the Identification of Relevant Species

### 9.1. Cultivation and stress tests

Today, the capability of microorganisms to withstand the deleterious environmental factors during a mission to the outer Solar System can only be determined with cultivable organisms, by performing stress tests. Depending on the species, the necessary cultivation time can be long, and take up to weeks and months, before results are available. This is especially the case for psychrophiles, organisms growing at low temperatures (−20°C to +15°C; Pulschen *et al.*, 2017) or halophiles, organisms growing at high salt concentrations of up to 30% NaCl (Pedrós-Alió *et al.*, 2000; Reid *et al.*, 2006; Burns *et al.*, 2007; Schneegurt *et al.*, 2012; Stan-Lotter and Frendrihan, 2015). Exposure to combined stresses can prolong the necessary incubation period even more. Systematic investigations on this topic are necessary to gain a deeper general understanding of the physiology of the microorganisms in a spacecraft assembly clean room, the composition of the microbial community, and the identification of potentially problematic species.

**Statement 6:** Although microbial strains from spacecraft assembly clean rooms and spacecraft from ESA missions are deposited in a publicly available culture collection, the majority of microbial strains isolated in the context of missions performed by NASA and other spacefaring nations cannot be obtained for basic research.

**Recommendation 5:** Establish and maintain an international culture collection for microorganisms from spacecraft and spacecraft assembly clean rooms, including those from non-ESA missions.

To identify microbial species that are problematic for icy moons, a systematic overview of typical microbial clean room inhabitants is necessary.

**Recommendation 6:**

(a) Systematically compile the available published data about the extremotolerance of clean room isolates with a focus on desiccation and radiation resistance, halophilic, oligotrophic (autotrophic), psychrophilic, (facultative) anaerobic growth.
(b) Investigate the desiccation and radiation resistance of representative spacecraft assembly clean room isolates, starting with organisms that are already available in culture collections.
(c) Collect new isolates representing a wide range of metabolisms from clean rooms and investigate in the same way as in (b) to get a broader basis for a first estimation of the abundance of problematic species.
(d) Obtain additional new insights into the molecular and cellular stress response mechanisms of potentially problematic species from isolates from recent Earth analog environments for icy moons such as polar regions, subglacial lakes, and deep sea or terrestrial brines. Perform laboratory experiments under subsurface icy moon conditions to assess their potential for survival and replication.

Table 6 gives a preliminary list of bacteria to be investigated. All of them are publicly available from culture collections, for example, the ESA Microbial Collection at the German Collection of Microorganisms and Cell Cultures (DSMZ; https://www.dsmz.de). The current list is conditioned by a series of assumptions and practical considerations based on its focus on planetary protection. It should be seen as a draft, and open to later inclusions, updates, and revisions, depending on input from other experts and advances in our understanding of the microbiology of these locations as well as our knowledge on the environmental conditions on the icy moons of the outer Solar System. The selection criteria are listed in the table: detected in spacecraft assembly clean rooms by cultivation, representing a broad range of physiological capabilities, for example, (facultative) growth under anaerobic conditions, growth at low temperature, growth in the presence of salts (chlorides and sulfates), radiation resistance, desiccation resistance and association with humans, long-term use as model organism in space, or as a proxy for total bioburden in the actual standard assays for planetary protection.

**Table 6.** Preliminary List of Bacteria to be Investigated

*Organism*	*Phylum*	*Potential concern*	*Temperature range*	*Culture collection*	*Reference*
(a) Previously detected in clean rooms and available from public culture collections					
*Bacillus pumilus*	Firmicutes	Spore-forming, UV resistant	Mesophile	DSM 30550	Moissl-Eichinger *et al.* (2012)
*Micrococcus luteus*	Actinobacteria	—	Mesophile	DSM 30505	Moissl-Eichinger *et al.* (2012)
*Staphylococcus aureus*	Firmicutes	Human pathogen	Mesophile	DSM 30501	Moissl-Eichinger *et al.* (2012)
*Staphylococcus epidermidis*	Firmicutes	Human pathogen	Mesophile	DSM 30936	Moissl-Eichinger *et al.* (2012)
*Clostridium perfringes*	Firmicutes	Strict anaeorobic	Mesophile	DSM 30770	Moissl-Eichinger *et al.* (2012)
*Paenibacillus phoenicis*	Firmicutes	Spore-forming, UV resistant	Mesophile	DSM 27463	Benardini *et al.* (2011)
*Brevundimonas* sp.	Proteobacteria	High survival under simulated Mars conditions	Mesophile	DSM 30891	Rettberg *et al.* (2006)
*Deinococcus phoenicis*	Deinococcus-Thermus	High radiation resistance, highly sensitive to NaCl	Mesophile	DSM 27173^a^	Vaishampayan *et al.* (2014)
(b) Previously detected in clean rooms and not available from public culture collections					
*Acinetobacter gyllenbergii 2P01AA*	Gammaproteobacteria	Highly oxidation resistant	Mesophile	—	Derecho *et al.* (2014)
(c) Not (yet) detected in clean rooms, of concern for icy moons					
*Halomonas halodenitrificans*	Proteobacteria	Halophile, psychrotolerant, facultative anaeorobic	5–32°C	ATCC 13511; DSM 735	Robinson and Gibbons (1952)
*Halorubrum lacusprofundi*	Euryarchaeota	Psychrophile, halophile	4–47°C	ATCC 49238; DSM 5037	Franzmann *et al.* (1988)
*Halanaerobium sehlinense*	Firmicutes	Anaerobic, halophile, alkalotolerant	20–50°C	DSM 25582	Abdeljabbar *et al.* (2013)
*Bacillus alcalophilus*	Firmicutes	Alkaliphile, halotolerant	10–40°C	ATCC 27557	Boyer *et al.* (1973)
*Thiobacillus ferrooxidans*	Proteobacteria	Facultative anaerobic, chemoautotroph, acidophile	18–37°C	DSM 585	and Trussell (1963)
*Planococcus halocryophilus*	Firmicutes	Psychrophile, halotolerant	−10–37°C	DSM 24743	Mykytczuk *et al.* (2012)
*Methanogenium frigidum*	Euryarchaeota	Psychrophile, anaeorobic	0–17°C	DSM 16458	Franzmann *et al.* (1997)
*Psychromonas antarcticus*	Proteobacteria	Anaeorob, psychrophile, low salt	2–22°C	DSM 10704	Mountfort *et al.* (1998)

^a^ Type strain.

ATCC, American Type Culture Collection; DSMZ, Deutsche Sammlung von Mikroorganismen und Zellkulturen.

The spacecraft assembly clean room isolates listed in Table 6A and B have been detected with the actual planetary protection standard assays that do not aim primarily at the identification and quantification of species relevant for icy moons. Some of these strains, namely any spore formers, might turn out to be problematic for icy moons. The list also includes taxa that have been shown to be active in icy moon analog environments (Table 6C), although they have not yet been isolated from clean rooms. Their inclusion is meant to further expand the physiological variability and better represent the total microbial diversity.

Based on the results to be expected from these investigations, a threshold for desiccation and radiation resistance of clean room microorganisms has to be defined, which allows the distinction of problematic species from nonproblematic species for icy moons.

**Statement 7:** The list of suggested test microorganisms should be seen as a draft, and open to later inclusions, updates, and revisions, depending on input from other experts and advances in our understanding of the microbiology of these locations as well as our knowledge on the environmental conditions on the icy moons of the outer Solar System.

### 9.2. Proposed test organisms

#### 9.2.1. Spacecraft assembly clean room isolates

##### 9.2.1.1. *Bacillus pumilus*

The Gram-positive aerobic rod-shaped endospore-forming bacteria of the genus *Bacillus* are the most widely represented organisms in soil and are frequently recovered in clean rooms or space hardware assembly facilities. Spores survive the heat-shock step and are detected with the planetary protection standard assay. Members of this species are highly resistant to extreme environmental conditions such as low or no nutrient availability, desiccation, irradiation, H_2_O_2_, and chemical disinfections (Nicholson *et al.*, 2000).

##### 9.2.1.2. *Paenibacillus phoenicis*

This novel Gram-positive motile endospore-forming aerobic bacterium was isolated from the NASA Phoenix lander assembly clean room that exhibits 100% 16S rRNA gene sequence similarity to two strains isolated from a deep subsurface environment (Bernadini *et al.*, 2011). *P. phoenicis* spores survive the heat-shock step and are detected with the planetary protection standard assay. The endospores of this rod-shaped bacterium are resistant to UV radiation up to 500 J/m^2^ (Bernardini *et al.*, 2011). Growth occurs between 21°C and 50°C, at pH 7.0–9.0, and in the presence of 5% NaCl. Optimum growth occurs at 37°C and at pH 7.0 (Bernadini *et al.*, 2011).

##### 9.2.1.3. *Micrococcus luteus*

*Micrococcus luteus* is a Gram-positive to Gram-variable nonmotile coccus saprotrophic bacterium that belongs to the family Micrococcaceae. *M. luteus* has been shown to survive in oligotrophic environments for extended periods of time, and has been frequently recovered from spacecraft assembly facilities (Rettberg *et al.*, 2006). Members of this species survive the heat-shock step and are detected with the planetary protection standard assay.

##### 9.2.1.4. *Staphylococcus aureus*

*Staphylococcus aureus* is a Gram-positive round-shaped facultative anaerobe that can grow in the absence of oxygen by fermentation or by using an alternative electron acceptor, and is frequently found in the human nose, respiratory tract, and skin (Masalha *et al.*, 2001). Since the microbial testing of the Viking spacecraft in 1977 (Puleo *et al.*, 1977), members of this species have been isolated and cultivated from samples obtained from clean room facilities. The genus *Staphylococcus* represents the most common nonspore-forming isolates recovered from clean room facilities (Smith *et al.*, 2017) and, due to their pathogenic nature, pose a threat to human exploration.

##### 9.2.1.5. *Staphylococcus epidermidis*

Similar to *S. aureus*, this strain is associated with human skin. It colonizes predominantly the axillae, head, and nares and is the most frequently isolated species from human epithelia (Otto, 2009). *Staphylococcus epidermidis* is a Gram-positive cocci nonspore-forming organism that usually grows aerobically; however, reports suggest that strictly anaerobic strains of *S. epidermidis* exist (Rowlinson *et al.*, 2006). Together with other species, the genus *Staphylococcus* represents the most common nonspore-forming isolates recovered from clean room facilities (Smith *et al.*, 2017).

##### 9.2.1.6. *Clostridium perfringens*

*Clostridium perfringens* is a Gram-positive rod-shaped anaerobic spore-forming pathogenic bacterium of the genus *Clostridium*. *C. perfringens* is omnipresent in nature and can be found as a normal component of decaying vegetation, marine sediment, the intestinal tract of humans, and other vertebrates, insects, and soil (Rood and Cole, 1991). Members of this species have been detected by molecular methods and cultivation assays in different spacecraft assembly facilities (Moissl *et al.*, 2007; Stiegelmeier *et al.*, 2009).

##### 9.2.1.7. *Brevundimonas* ssp.

The genus *Brevundimonas* comprises Gram-negative short rods that are 1 to 4 μm long and 0.5 μm in diameter. They grow aerobically between 30°C and 37°C, no growth occurs at 4°C, and no autotrophic growth occurs with H_2_ (Segers *et al.*, 1994). Although this genus is nonspore forming, previous investigations uncovered that representative strains survive high doses of gamma radiation and frequent freeze–thaw cycles (Dartnell *et al.*, 2010).

##### 9.2.1.8. *Acinetobacter gyllenbergii* 2P01AA

Gram-negative gammaproteobacteria of the genus *Acinetobacter* are common soil organisms where they contribute to the degradation of aromatic compounds. In hospitals, other *Acinetobacter* species are a source of infections in debilitated patients. During the Mars Phoenix lander assembly, several hydrogen peroxide-resistant *Acinetobacter* strains have been isolated (Derecho *et al.*, 2014). Among these, *A. gyllenbergii* 2P01AA exhibited the highest resistance.

#### 9.2.2. Examples of species relevant for icy moons

##### 9.2.2.1. *Halomonas halodenitrificans*

Gram-negative coccus, 0.5 μm in diameter, occurring singly or in pairs. Sodium chloride is necessary for growth and the organism dies at or <2.2%. The coccus grows optimally in medium containing between 4.4% and 8.8% sodium chloride; above 8.8%, the length of the lag phase increases and the rate of growth decreases; however, growth is observed up to 24% (Robinson and Gibbons, 1952). The strains grow under aerobic or facultatively anaerobic conditions.

##### 9.2.2.2. *Halorubrum lacusprofundi*

This strain was isolated from deep lake, Antarctica, and first described by Franzmann *et al.* in 1988. Unlike other halobacteria, the strains grow at 4°C, although very slowly. Sodium chloride concentrations range from 1.5 mol/L to saturation. The strain is not proteolytic, does not produce acids from sugars, but utilizes a wide range of carbon sources including sugars, alcohols, and organic acids for growth.

##### 9.2.2.3. *Halanaerobium sehlinense*

This strictly anaerobic extremely halophilic Gram-positive rod-shaped bacterium was isolated from the hypersaline (20% NaCl) surface sediments of Sehline Sebkha in Tunisia (Abdeljabbar *et al.*, 2013). *Halanaerobium sehlinense* is nonspore forming, nonmotile, appearing singly or in pairs, or occasionally as long chains and measured 0.5–0.8 μm by 3–10 μm. The organism is able to ferment glucose, galactose, fructose, glycerol, mannose, maltose, ribose, pyruvate, and sucrose. There are no previous reports about the resistance of this strain against desiccation and radiation.

##### 9.2.2.4. *Bacillus alcalophilus* subsp. halodurans

*Bacillus alcalophilus* grows best in alkaline medium (pH between 8 and 10) with mediocre growth at pH 7. The strain is Gram-positive, motile, rod, and spore forming as well as facultatively anaerobic (Boyer *et al.*, 1973). Growth can be observed up to 15% NaCl, but higher concentrations need to be evaluated.

##### 9.2.2.5. *Acidothiobacillus ferrooxidans*

*Acidothiobacillus ferrooxidans* is a chemolithotrophic autotroph able to obtain energy from the oxidation of ferrous iron or inorganic sulfur compounds (Tuovinen and Kelly, 1973*). A. ferrooxidans* is generally assumed to be an obligatory aerobic organism. However, under anaerobic conditions, ferric iron can replace oxygen as an electron acceptor for the oxidation of elemental sulfur (Bauermeister *et al.*, 2014).

##### 9.2.2.6. *Planococcus halocryophilus*

This aerobic Gram-positive motile coccoid bacterial strain was isolated from permafrost active-layer soil collected from the Canadian High Arctic (Mykytczuk *et al.*, 2012). Cells grow aerobically, are nonspore-forming, motile cocci, 0.8–1.2 μm in diameter, and occur singly or in pairs. *Planococcus halocryophilus* is capable of growth over a broad temperature range, including subzero growth (below −10°C to 37°C), and at relatively high salinity (0–19% NaCl).

##### 9.2.2.7. *Methanogenium frigidum*

*Methanogenium frigidum* was isolated from the perennially cold anoxic hypolimnion of Ace Lake in the Vesfold Hills of Antarctica. The cells were psychrophilic, exhibiting most rapid growth at 15°C, and no growth at temperatures >18°C to 20°C. The cells were irregular nonmotile coccoids (diameter 1.2–2.5 μm) that occurred singly and grew by CO_2_ reduction by using H_2_ as a reductant (Franzmann *et al.*, 1997). Organisms are halotolerant and obligate psychrophile.

##### 9.2.2.8. *Psychromonas antarcticus*

This Gram-negative rod- to oval-shaped aero-tolerant anaerobic bacterium was isolated from an anaerobic enrichment inoculated with sediment taken from below the cyanobacterial mat of a high-salinity pond near Bratina Island on the McMurdo Ice Shelf, Antarctica (Mountfort *et al.*, 1998). Temperature range for growth, 2°C to <22°C, with optimum at 12°C.

None of these problematic species has been investigated on their desiccation and radiation resistance in detail. It is, therefore, unclear at the moment whether they could survive a spaceflight to an icy moon and whether they could proliferate in the icy moons' subsurface oceans.

### 9.3. Functional genomics

(Gene-resolved) metagenomics (Zhang *et al.*, 2010), single cell genomics, and other omics technologies have led to sophisticated systems biology approaches, which could give insights into microbial metabolism, microbial community functions, and their interactions with the environment (Hedlund *et al.*, 2014; Cowan *et al.*, 2015; Bashir *et al.*, 2016; Minich *et al.*, 2018). A functional genomics approach can be a suitable cultivation-independent method to identify problematic species and their metabolic function from clean room metagenome data. To understand the microbial physiology of microorganisms in a certain environment, it is necessary to consider not only the presence of specific genes but also their gene products, mRNA, proteins, and metabolites (Zhang *et al.*, 2010; Manor *et al.*, 2014).

The usefulness of the identification and quantification of indicator sequences/genes from metagenome data with respect to physiological capabilities pointing to problematic species should be tested by direct comparison with cultivation and stress test data obtained from comparable spacecraft assembly clean room samples. First steps to identify candidate genes/sequences responsible for the unusual resistance of some *Bacillus* species isolated from spacecraft assembly clean rooms to UV radiation and oxidative agents have already been performed (Tirumalai *et al.*, 2013). Other examples are to investigate the DNA Damage Regulon with a radiation/desiccation response motif identified in highly radiation and desiccation-resistant bacteria (Anaganti *et al.*, 2017) or genes and gene products necessary for other relevant metabolic processes like the potential for H_2_ oxidation or H_2_ production by hydrogenases (Brazelton *et al.*, 2012).

However, this type of basic investigations takes time and requires research projects that are not coupled to specific space missions with tight schedules.

**Recommendation 7:** Screen clean room genome-resolved metagenomic data to identify microbial genes/sequences that can be used as indicators for problematic species (desiccation resistance, radiation resistance, halophilic, anaerobic, and psychrophilic growth in low biomass microbial communities).

**Recommendation 8:** Verify whether the comparison of stress test results of clean room isolates and the identified microbial genes/sequences for specific physiological capabilities allows the assessment of the physiological potential of the clean room microbiota qualitatively and quantitatively.

## 10. Short-term Research Activities for the Development of New Planetary Protection Assays for the Next Icy Moons Missions

As already mentioned due to time constraints, cultivation assays for all potentially problematic species are not suitable for routine clean room monitoring and planetary protection bioburden measurements during assembly, integration, and testing activities.

For near future missions to the outer Solar System, there is a need for new fast assays whose results are indicative for problematic species and take into account the most challenging environmental factors (*i.e.*, desiccating conditions and exposure to high doses of radiation). Investigations were already initiated in 2001 by Chen *et al.* In their study, clean room samples were exposed to a radiation dose of 10 kGy. No surviving microorganisms could be found, but 16S rDNA fragments could still be amplified. A systematic investigation is necessary as a basis for the definition of a new bioburden assay and corresponding new bioburden limits for problematic species.

**Recommendation 9:** Define and evaluate a new planetary protection bioburden assay for icy moons based on preselection steps in terms of desiccation and radiation exposure.

**Recommendation 10:** Define bioburden limits for missions to icy moons based on the knowledge about the problematic species in clean rooms, their physiological capabilities, and the to be defined new bioburden assay.

**FIG. 4.**
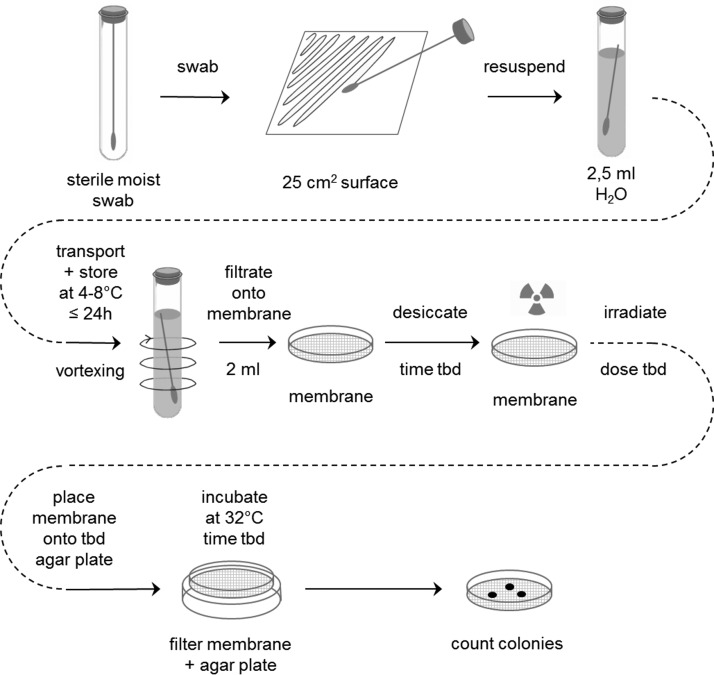
Suggested new standard assay for problematic species.

One first practical approach for the final assay could be the concentration of microorganisms from clean room and spacecraft samples on membrane filters, followed by a desiccation step under controlled conditions and an irradiation step with ionizing radiation (duration of desiccation and dose of radiation to be defined). As already described, space radiation, consisting of sparsely and densely ionizing radiation, is very complex and cannot be reproduced on Earth. Therefore, the use of X-rays as a proxy for space radiation is recommended, even if the induced cell damages are not totally identical to those resulting from the absorbance of HZE particles or the exposure to space radiation. An example of a possible assay for swab samples is shown in Fig. 4.

## 11. Summary

To define new planetary protection bioburden assays relevant for icy moons, systematic investigations should aim to (i) identify potential problematic species for icy moons in clean rooms by applying a combination of cultivation-dependent and cultivation-independent methods, (ii) develop/optimize methods for the discrimination between dead and alive microorganisms, (iii) determine the resistance of representative clean room contaminants to simulated mission parameters (*e.g.*, vacuum and ionizing radiation), (iv) determine the resistance of the potential problematic species to the environmental conditions of the mission target (*e.g.*, radiation, salts, and low temperature), and (v) develop suitable new planetary protection assays for icy moons and derive threshold values (*e.g.*, irradiation doses and desiccation duration) from (iii) and (iv) for problematic species that can serve as proxies for all potential problematic species from clean rooms.

## Footnotes

^*^ The statements are numbered in the order they appear in the text. They summarize general findings as well as opinions of the PPOSS team as the basis of the recommendations given in this report.

^†^ Recommendations are numbered to facilitate referencing. The order does not indicate the importance or timeframe.

## Notes

Associate Editor: Christopher McKay

## Abbreviations Used

ATP: adenosine triphosphate
CFUs: colony-forming units
COSPAR: Committee on Space Research
ECSS: European Cooperation for Space Standardization
EURECA: European Retrievable Carriere
HZE: high (H) atomic number (Z) and energy (E)
LDEF: Long Duration Exposure Facility
PMA: propidium monoazide
PPOSS: Planetary Protection of the Outer Solar System
RAD: Radiation Assessment Detector

## Appendix 1

The Planetary Protection of Outer Solar System consortium (http://pposs.org/)

- German Aerospace Center (DLR), Köln, Germany—Petra Rettberg, Stefan Leuko, Elke Rabbow
- Science Connect–European Science Foundation (ESF)—Strasbourg, France (Coordinator) Nicolas Walter, Patricia Cabezas
- Committee of Space Research (COSPAR), Montpellier, France—Jean-Loius Fellous, Alissa Haddaji, Gerhard Kminek
- Eurospace, Paris, France—Jean-Charles Treuet
- Imperial College, London, United Kingdom—Mark Sephton
- National Institute for Astrophysics (INAF), Florence, Italy—John Brucato
- Space Technology Ireland Ltd., Ireleand—Susan McKenna-Lawlor

Expert

TASI, Italy—Diana Margheritis

International Partner

China Academy of Space Technology, China.

International Observer

Office of the Space Studies Board of the National Research Council office, USA

## Appendix 2

Participants of the Biological and Organic Contamination Prevention Workshops, January 23–27, 2017 at DLR in Köln, Germany, and April 10–12, 2017 at INAF in Firenze, Italy.

André Antunes Edge Hill University, United Kingdom
Berger, Thomas DLR, Germany
Canham, John Orbital ATK, USA
Collins, Geoffrey Wheaton College, USA
Cullen, David Cranfield University, United Kingdom
Dworkin, Jason GSFC, USA
Francis, Karen Olson The Open University, United Kingdom
Funke, Oliver DLR, Germany
Grasset, Olivier Nantes University, France
Horneck, Gerda DLR, Germany
Margheritis, Diana TASI, Italy
Marteinsson, Vigo Matis, Iceland
Martin, Willliam University of Düsseldorf, Germany
Pearce, David Northumbria University, United Kingdom
Pearson, Victoria The Open University, United Kingdom
Raulin, Francois LISA/UPEC-CNRS, France
Saunders, Mark Independent consultant for the National Academies of Sciences (NAS), USA
Scharge, Yvonne Max Plank Institute for Solar System Research, Germany
Spry, Andy SETI, USA
Smith, David Space Studies Board, USA
Steininger, Harald Max Plank Institute for Solar System Research, Germany
Watson, Jonathan IC, United Kingdom
Yano, Hajime ISAS/JAXA, Japan
